# Association between neighborhood safety and overweight status among urban adolescents

**DOI:** 10.1186/1471-2458-9-289

**Published:** 2009-08-11

**Authors:** Dustin T Duncan, Renee M Johnson, Beth E Molnar, Deborah Azrael

**Affiliations:** 1 Department of Society, Human Development and Health, Harvard School of Public Health, Boston, MA, USA; 2 Harvard Youth Violence Prevention Center, Harvard School of Public Health, Boston, MA, USA; 3 Department of Health Policy and Management, Harvard School of Public Health, Boson, MA, USA; 4 Department of Community Health Sciences, Boston University School of Public Health, Boson, MA, USA

## Abstract

**Background:**

Neighborhood safety may be an important social environmental determinant of overweight. We examined the relationship between perceived neighborhood safety and overweight status, and assessed the validity of reported neighborhood safety among a representative community sample of urban adolescents (who were racially and ethnically diverse).

**Methods:**

Data come from the 2006 Boston Youth Survey, a cross-sectional study in which public high school students in Boston, MA completed a pencil-and-paper survey. The study used a two-stage, stratified sampling design whereby schools and then 9^th^–12^th ^grade classrooms within schools were selected (the analytic sample included 1,140 students). Students reported their perceptions of neighborhood safety and several associated dimensions. With self-reported height and weight data, we computed body mass index (BMI, kg/m^2^) for the adolescents based on CDC growth charts. Chi-square statistics and corresponding *p*-values were computed to compare perceived neighborhood safety by the several associated dimensions. Prevalence ratios (PRs) and 95% confidence intervals (CI) were calculated to examine the association between perceived neighborhood safety and the prevalence of overweight status controlling for relevant covariates and school site.

**Results:**

More than one-third (35.6%) of students said they always felt safe in their neighborhood, 43.9% said they sometimes felt safe, 11.6% rarely felt safe, and 8.9% never felt safe. Those students who reported that they rarely or never feel safe in their neighborhoods were more likely than those who said they always or sometimes feel safe to believe that gang violence was a serious problem in their neighborhood or school (68.0% vs. 44.1%, *p *< 0.001), and to have seen someone in their neighborhood assaulted with a weapon (other than a firearm) in the past 12 months (17.8% vs. 11.3%, *p *= 0.025). In the fully adjusted model (including grade and school) stratified by race/ethnicity, we found a statistically significant association between feeling unsafe in one's own neighborhood and overweight status among those in the Other race/ethnicity group [(PR = 1.56, (95% CI: 1.02, 2.40)].

**Conclusion:**

Data suggest that perception of neighborhood safety may be associated with overweight status among urban adolescents in certain racial/ethnic groups. Policies and programs to address neighborhood safety may also be preventive for adolescent overweight.

## Background

Adolescent overweight is a major public health priority because of the immediate and long-term health risks it poses and because of its rapidly increasing prevalence. Specifically, adolescent overweight is associated with numerous deleterious chronic health consequences in adolescence, such as hypertension [1-3], type 2 diabetes [1,3], and asthma [1,3-5], and in adulthood, such as hypertension [1,3,6], type 2 diabetes [1,3,7], coronary heart disease [1,7,8], and certain cancers [1,7-9]. Trend data indicate that overweight among adolescents has been dramatically increasing [10-12]. The prevalence of being overweight among 12–19 year olds, for example, has more than doubled in recent decades; going from 6.1% in 1971–1974 to 15.5% in 1999–2000 [11].

In an effort to thwart the overweight epidemic, research attention has increasingly been placed on social and environmental factors that give rise to overweight [10,13,14]. Neighborhood safety may be an important contributor to overweight, as it has been theorized that fear of violence and crime in the immediate social environment is a barrier to physical activity and a facilitator of sedentary behavior (two well-established predictors of overweight). Several studies among adolescents have shown an inverse association between neighborhood safety and physical activity [15-28], and others have demonstrated a positive association between neighborhood safety and sedentary behavior [17,18]. The few studies that have examined neighborhood safety as a predictor of overweight among adolescents have yielded inconsistent findings, with some finding a statistically significant inverse association [29,30] and others not having found such evidence [18,19,31,32]. These inconsistencies may be due to differences across study populations, in the measurement of neighborhood safety, and/or in the categorization of overweight status.

There are limitations with and gaps in the extant literature worth noting. First, most cross-sectional studies that examine neighborhood safety and overweight status among adolescents have used odds ratios for the studied associations. However, prevalence ratios are more appropriate because they provide a more valid parameter estimate when the prevalence of the outcome is common while the odds ratio is likely to overestimate the effect when there is a relatively high disease prevalence [33-36]. Given the high prevalence of adolescent overweight [10,11,37,38], prevalence ratios probably should be estimated when examining correlates of adolescent overweight. Second, few studies of neighborhood safety and adolescent overweight have been conducted among racially and ethnically diverse samples [19,31,32]. Generalizability of the previous research findings is therefore limited. Third, none of the existing studies in this area have sought to evaluate the accuracy of the measure of perceived neighborhood safety. Threats to validity (e.g. misclassification of neighborhood safety) would likely result in an underestimation of effects due to non-differential misclassification bias.

The primary aim of the present study was to examine the association between neighborhood safety and overweight status among a representative sample of public high school students in Boston, MA. This study improves on earlier studies by using prevalence ratios as the parameter estimate, with a racially and ethnically diverse sample and by assessing the validity of perceived neighborhood safety (our secondary objective). To achieve the latter, we examined associations between perceived neighborhood safety and associated dimensions, including beliefs about gang activity and witnessed violence. Our evaluation of the validity of neighborhood safety is a novel contribution to the literature.

## Methods

### Sample Selection

Data for this investigation come from the 2006 *Boston Youth Survey *(BYS), a biennial survey of high school students (9^th^–12^th ^graders) in selected Boston Public Schools. We used a two-stage, stratified random sampling strategy. The first sampling frame consisted of all 34 high schools in the Boston Public Schools system. Thirty schools were randomly selected for the survey, with a probability of selection proportional to each school's enrollment size. Eighteen schools agreed to participate.

Among the participating schools, we generated a numbered list of unique homeroom classes within each school. First, classrooms comprised of students with severe cognitive disabilities were excluded. Next, classrooms were stratified by grade, and then randomly selected for survey administration within each grade. Those classrooms that listed fewer than five students were skipped and the next randomly selected classroom was chosen. Selection continued until the total number of students to be surveyed ranged from 100–125 per school. In the two selected schools with total enrollments close to 100, all students in the school were invited to participate.

### Data Collection

The BYS data collection instrument was developed by study staff. The instrument covered a range of topics (e.g., health behaviors, use of school and community resources, and indicators of positive youth development), and had a particular emphasis on violence. Items addressing violence inquired about aggressive behavior, victimization and assault, witnessed violence, fear of violence, and weapon carrying. The paper-and-pencil survey was administered in classrooms by trained staff in the spring of 2006. Survey administrators completed a brief training program prior to going into the schools. All personnel underwent training in the ethical treatment of human subjects at the Harvard School of Public Health.

Surveys were not marked with any information that could identify an individual. Passive consent was sought from students' parents prior to survey administration. Any student whose parent sent back a form denying permission for the student to participate in the survey was not given one; this was the case for less than 1% of students. Survey administrators read an introduction and the informed consent statement prior to distributing the survey. Seventy of the 1,323 invited students (5.3%) declined to participate. Survey administrators remained in the room and were available to answer questions throughout the 50 minutes allotted for the survey. The Office of Human Research Administration at the Harvard School of Public Health approved all procedures for this research project.

### Study Variables

The primary outcome variable was whether adolescents were overweight or at-risk for becoming overweight. To create this variable, we first calculated body mass index (BMI) using respondents' answers to items on height and weight, i.e., weight in kilograms divided by the square of height in meters. We then used BMI to classify respondents as underweight, healthy weight, at-risk for being overweight, or overweight, using age- and sex-specific BMI cut-offs based on Centers for Disease Control and Prevention (CDC) growth charts from the year 2000 [39]. Adolescents who were at or above the 85^th ^percentile were classified at being at-risk for overweight, while those at or above the 95^th ^percentile were classified as overweight. The four-level weight classification variable was subsequently reduced to a dichotomous variable of "at-risk/overweight" and "underweight/normal".

The primary predictor variable was adolescents' perceptions of neighborhood safety. We assessed it with a question designed to capture a global perception of neighborhood safety, i.e., "Do you feel safe in your neighborhood?". The item had the following four response options: always, sometimes, rarely, and never, and was dichotomized (i.e., always/sometimes and rarely/never) for analyses. To assess the validity of perceptions of neighborhood safety, we evaluated the association between perceptions of safety with: (1) beliefs about the seriousness of gang activity in the neighborhood or school, (2) having witnessed someone in the neighborhood being attacked with a weapon (other than a gun) in the past 12 months, and (3) having witnessed someone being physically attacked (i.e. punched, kicked, choked, or beaten up) in the neighborhood in the past 12 months.

Covariates included: age (≤14–≥18 years), grade level (9^th^–12^th^), sex (male, female), nativity (U.S. born, foreign-born), and race/ethnicity. To assess race, students were asked to indicate if they were: White; American Indian or Alaska Native; Asian; Black or African American; Native Hawaiian or Other Pacific Islander; Some Other Race, or any combination of those options. We combined Hispanic/Latino ethnicity and race to create a race/ethnicity variable with the following four levels: (1) Hispanic/Latino; (2) non-Hispanic Black/African American; (3) non-Hispanic White; and (4) Other, which includes non-Hispanic bi- or multi-racial students, Asians, American Indians, and other racial groups. To preserve respondent confidentiality, statistics by sub-groups within the "Other" category are not reported.

### Statistical Analysis

Although there were 1,253 surveys collected in the 18 schools, the surveys of 38 students (3%) were excluded from data analysis: 35 because they left at least 80% of the items unanswered, and 3 because of erratic answering patterns. Of the remaining 1,215 respondents, 75 were restricted from the analysis sample because they did not answer the items on height and/or weight (and therefore we could not create a BMI variable for them) or because they did not answer the perceived neighborhood safety item. This resulted in an analytic sample size of 1,140.

Data analysis was performed using SAS statistical software version 9.1.3 [40]. First, we generated descriptive information for socio-demographic characteristics of the sample, perceived neighborhood safety, and correlates of perceived neighborhood safety (i.e., beliefs about gang violence, witnessed a physical attack with a weapon other than a gun in the past 12 months, and witnessed someone being physically assaulted in the past 12 months). Next, chi-square statistics and corresponding *p-v*alues were computed to assess group differences in overweight status by socio-demographic factors, and in perceived neighborhood safety by the associated dimensions. We implemented a multiple comparisons test for proportions using the methods established by Zar [41] for the socio-demographic factors; this was accomplished by using the COMPPROP macro in SAS [42].

We examined whether the association between neighborhood safety and overweight/at-risk for overweight status varied by sex by fitting sex-stratified models because we hypothesized different mechanisms for adolescent boys and girls. Prevalence ratios (PRs) and 95% confidence intervals (CI) were calculated to examine the bivariate association between perceived neighborhood safety and the prevalence of being overweight or at-risk for becoming overweight. We then fit a multiple regression model, in which we adjusted for socio-demographic factors as appropriate based on group differences. If associated with both the predictor and outcome variables, we included them in the final model. In the final model, we controlled for clustering of students within schools by fitting a generalized estimating equation (GEE) model, with the cluster variable specified as school. Because overweight was analyzed as a dichotomous variable, we specified a binomial response distribution. We used a log link function to relate the expected value of the outcome to the predictor because the data are cross-sectional [43,44]. GEE models were fit using PROC GENMOD in SAS, with school specified as the subject in the REPEATED statement. Listwise deletion was used in regression analyses, i.e., individuals with missing data on any of the covariates were excluded from regression models. Statistical significance was determined by 95% CIs.

## Results

More than half of the respondents in the analysis sample (n = 1,140) were female (57.8%), and 46.5% were non-Hispanic Black (Table 1). The mean age was 16.3 years (SD = 1.3). The majority of respondents (54.1%) were in the normal BMI range, 1.1% were underweight, 17.5% were at-risk for becoming overweight, and 27.3% were overweight. Almost one-third of the students were born outside of the U.S. (29.8%).

**Table 1:** Socio-demographic characteristics of respondents by overweight status

	**Total** **n = 1,140** **n (%)**	**Normal or underweight** **n = 629** **n (%)**	**At risk or overweight** **n = 511** **n (%)**	***χ*^2 ^Statistic** **(*p*-value)**
**Race/Ethnicity**				10.62 (0.014)
Hispanic	335 (30.0)	169 (27.4)	166 (33.2)	
Black, non-Hispanic	520 (46.5)	283 (45.8)	237 (47.4)	
White, non-Hispanic	145 (13.0)	88 (14.2)	57 (11.4)	
Other^a^	118 (10.6)	78 (12.6)	40 (08.0)	
				
**Sex**				1.02 (0.311)
Male	481 (42.2)	257 (40.9)	224 (43.8)	
Female	659 (57.8)	372 (59.1)	287 (56.2)	
				
**Nativity**				3.54 (0.060)
US Born	788 (70.2)	420 (67.9)	368 (73.0)	
Foreign Born	335 (29.8)	199 (32.2)	136 (27.0)	
				
**Grade**				8.08 (0.044)
Ninth	325 (29.0)	162 (26.1)	163 (32.7)	
Tenth	293 (26.1)	162 (26.1)	131 (26.3)	
Eleventh	332 (29.6)	202 (32.5)	130 (26.3)	
Twelfth	171 (15.3)	96 (15.4)	75 (15.0)	

*Note*. Totals may not sum to 1,140 due to missing data.

^a ^The Other category includes non-Hispanics students who chose two or more races, or who were Asian, American Indian/Alaska Native, Native Hawaiian or Other Pacific Islander. Information about these groups are not reported separately to preserve confidentiality.

Although there was no difference in overweight/at-risk for overweight status by sex, age or nativity, there were statistically significant group differences in weight status by race/ethnicity and by grade (Table 1). One-half of the Hispanic students were at-risk or overweight (49.6%), compared to 45.6% of Blacks, 39.3% of Whites, and 33.9% of those in the Other race/ethnicity group. Ninth graders were the most likely to be at-risk or overweight (50.2%), following by tenth graders (44.7%), twelfth graders (43.9%), and eleventh graders (39.1%). Multiple comparisons tests for proportions showed that the only statistically significant pairwise differences were between Hispanics and students in the Other race/ethnicity group, and between the ninth and eleventh grade students.

More than one-third (35.6%) of students said they always felt safe in their neighborhood, 43.9% said they sometimes felt safe, 11.6% rarely felt safe, and 8.9% never felt safe. There were no statistically significant differences in the proportion of students who said they rarely or never feel safe in their neighborhood by sex or age. There were, however, statistically significant differences in perceptions by race/ethnicity (*p *< 0.001), nativity (*p *< 0.05), and grade level (*p *< 0.015). Blacks, immigrants and twelfth graders were significantly more likely than others to report that they rarely or never felt safe in their neighborhoods. One-quarter of Blacks reported they rarely or never felt safe in their neighborhood, compared to 18.2% of Hispanics, 17.8% of Whites, and 9% of students in the Other race/ethnicity group. Compared to 18.7% of US-born students, nearly one-quarter of foreign-born students rarely or never felt safe in their neighborhood.

Those students who reported that they rarely or never feel safe in their neighborhoods were more likely than those who said they always or sometimes feel safe to believe that gang violence was a serious problem in their neighborhood or school, and to have seen someone in their neighborhood attacked with a weapon (other than a firearm) in the past 12 months (Table 2). This finding suggests that those who felt unsafe had more exposure to neighborhood violence. Adolescents' who reported that they rarely or never feel safe in their neighborhoods were no more likely than those who said they always or sometimes feel safe to have seen someone in their neighborhood physically assaulted in the past 12 months, indicating that seeing fights may be a frequent occurrence for adolescents regardless of neighborhood.

**Table 2:** Perceptions of neighborhood safety by dimensions of neighborhood violence

	**Believe there is a lot of gang violence in the neighborhood or school**	**Saw someone attacked with a weapon (other than a gun) in the neighborhood^b^**	**Saw someone physically assaulted in the neighborhood^b^**
**Rarely or never feel safe**	68.0%	17.8%	25.0%
**Always or sometimes feel safe**	44.1%	11.3%	22.3%
***p*-value^a^**	<0.001	0.025	0.463

^a^*p *for chi-squared test for perceived neighborhood safety by the associated dimensions.

^b ^Time referent is in the past 12 months.

Next we ran regression models to estimate the effect of perceived neighborhood safety on overweight/at-risk for overweight status. Although the models were initially run stratified by sex, we present the pooled results because prevalence ratios were similar across both sexes. The first model, of the crude association between perception of neighborhood safety and overweight status, indicates that those who rarely or never felt safe in their neighborhoods were 1.21 times more likely to be at-risk for overweight or overweight as compared to those who always or sometimes felt safe in their neighborhoods (95% CI: 1.05, 1.40). Because race/ethnicity and grade level were associated with overweight status and with neighborhood safety, we adjusted for these factors in a second model; the magnitude of the association is attenuated by about 4% (PR = 1.16, 95% CI: 0.99, 1.35). In the third final model, we adjusted for race/ethnicity, grade level, and controlled for clustering of observations by school. Although we found that those who said they rarely or never feel safe in their neighborhoods were 1.16 times more likely to be overweight or at-risk for overweight as compared to adolescents who said they always or sometimes feel safe, this result was not statistically significant (95% CI = 0.97, 1.38). We reran the series of regression models stratifying by race/ethnicity since 1) we were interested in parameter estimates by race/ethnicity, 2) race/ethnicity was associated with both the independent variable and outcome variable, and 3) the distribution of students by race/ethnicity varied substantially by school (Table 3). Interestingly, those in the Other race/ethnicity group who reported rarely or never feeling safe in their neighborhood were more likely to be at-risk or overweight in the fully adjusted model (PR = 1.56, 95% CI: 1.02, 2.40).

**Table 3:** Prevalence ratios (and 95% confidence intervals) estimating overweight status among students who rarely or never feel safe in their neighborhood

	**Model 1** **PR (95% CI)**	**Model 2^a^** **PR (95% CI)**	**Model 3^b^** **PR (95% CI)**
**Hispanic**	1.24 (0.98, 1.59)	1.16 (0.91, 1.49)	1.16 (0.89, 1.50)
**Black, Non-Hispanic**	1.09 (0.88, 1.34)	1.10 (0.99, 1.36)	1.10 (0.90, 1.34)
**White, Non-Hispanic**	1.42 (0.82, 2.46)	1.25 (0.71, 2.18)	1.23 (0.85, 1.79)
**Other**	1.54 (0.90, 2.64)	1.58 (0.91, 2.73)	1.56 (1.02, 2.40)

*Note*. PR = Prevalence Ratio. CI = Confidence Interval. Reference Category = Students who always or sometimes feel safe in their neighborhood

^a ^Adjusted for grade level using a generalized linear regression model.

^b ^Adjusted for grade level, using a generalized estimating equation model that accounted for clustering by school.

## Discussion

This population-based study of public high school students in Boston, MA adds to the accumulating body of evidence on the association between neighborhood safety and adolescent overweight. Our data suggest that feeling unsafe in one's neighborhood may be associated with an increased risk for overweight. However, this finding was only statistically significant among those within the Other race/ethnicity group. The Other group is comprised mainly of Asians and South Asians (65%), but also includes non-Hispanic bi- or multi-racial students, American Indians and Alaska Natives, Native Hawaiians and Other Pacific Islanders, and other students whose race did not fit into any of the other categories (e.g. those who were Guyanese, Belizean, or Brazilian). Thirty-eight percent of the students in the Other category were immigrants, compared to 34% of Hispanics, 31% of Blacks, and 12% of Whites.

Our findings are consistent with some previous studies that have found an association between neighborhood safety and adolescent overweight status [29,30], but inconsistent with others that did not find an association [18,19,31,32]. Few studies have examined the association between neighborhood safety and adolescent overweight stratified by race/ethnicity [32]. Our findings highlight the importance of considering the moderating effects of race/ethnicity in the association between neighborhood safety and overweight status. The magnitude of the association varied by race/ethnicity and the association was statistically significant for only one racial/ethnic group. We were surprised that the association held for only those within the Other race/ethnicity group; students in the Other group were the least likely to feel unsafe in their neighborhoods and were the least likely to be at-risk or overweight. We expected to find associations among Blacks and Hispanics, given that Black and Hispanic adolescents report particularly high levels of neighborhood violence exposure [45-49] and given that these adolescents are more likely to be overweight compared to Whites [10-12,37,38]. Interestingly, the magnitude of the association found among those within the Other race/ethnicity group was weaker in our study compared to the two other studies finding an association [29,30], perhaps a result of using prevalence ratios as the measure of effect (since these studies relied on odds ratios which likely produce inflated effect estimates when there is a relative high disease prevalence [33-36], such as adolescent overweight) [10,11,37,38] and/or because both studies found associations with traffic-related neighborhood safety (though the studies examined other safety-related variables). Because we examined general feelings of neighborhood safety, we can only speculate on which of the multiple aspects of neighborhood safety (e.g. violence/crime, traffic and road hazards, neighborhood disorder) might influence overweight status, but our interviews with students during the pilot testing phase strongly suggest that neighborhood violence is their primary neighborhood safety concern.

There are several pathways by which neighborhood safety might be related to adolescent overweight. One possible interpretation of our findings is that adolescents' concerns about neighborhood safety might decrease their willingness to engage in outdoor physical activity (e.g. walking and playing sports in their neighborhood), promote their use of non-ambulatory transportation options (e.g. use of buses, subways, and automobiles), and/or encourage sedentary behaviors (e.g. television watching, playing computer games, and playing video games in the home), all of which could contribute to being overweight [50,51]. Residing in an unsafe neighborhood might also increase stress (causing a release of cortisol) and result in overweight [52-55]. Evidence indicates that exposure to neighborhood violence, which is a potentially chronic traumatic stressor, is associated with increased cortisol secretion in adolescents [56].

Additionally, since we do not know whether students who perceived their neighborhoods to be unsafe *actually *live in unsafe neighborhoods and we are not aware of any study in this area that has conducted a validity check of perceptions of neighborhood safety, we explored whether students who reported feeling unsafe were more likely to have experienced neighborhood violence. We found that those students who felt unsafe in their neighborhoods were more likely than those who felt safe to believe that gang violence was a serious problem in their neighborhood or school and to have seen someone in their neighborhood attacked with a weapon (other than a firearm) in the past 12 months. However, they were not significantly more likely to have seen someone beaten up in their neighborhood in the past 12 months. These findings potentially indicate that witnessing a physical assault may not be a salient dimension of perceived neighborhood safety for adolescents, while neighborhood gang violence and seeing someone in their neighborhood assaulted with a weapon are important aspects of perceived neighborhood safety for them. This is an important contribution to the literature.

There is a need for additional research to clarify the role that neighborhood safety (including neighborhood violence) plays in the adolescent overweight epidemic and to understand salient aspects of perceptions of neighborhood safety. Perceived neighborhood safety is a complex, multidimensional psychosocial construct. Because there is no consensus on the definition of neighborhood safety in health research, qualitative research to explore the dimensions of neighborhood safety and to determine which dimensions of safety are most salient to adolescents at risk of overweight is warranted; this may vary by race/ethnicity. As all of the studies examining neighborhood safety and overweight in adolescents were cross-sectional, researchers should examine this association with prospective cohort designs. In addition, experimental research, e.g., cluster randomized trials (e.g. where neighborhoods might be randomly assigned to an intervention that improves safety) and natural experiments (e.g. new policies promoting police presence in certain neighborhoods to enhance safety), could be conducted (as they are the strongest evidence for temporality) to understand the effects of neighborhood safety on adolescent overweight. Neighborhood environmental interventions [57] and residential mobility experiments [58-60] hold promise to reduce the prevalence of adolescent overweight. Beyond examining perceived neighborhood safety, research can also examine objective neighborhood safety (e.g. crime statistics to ascertain one's proximity to neighborhood violence) in relation to overweight. Including both subjective and objective reports of neighborhood safety in the same study, although these concepts are likely interconnected, can be beneficial. Each measure might capture distinct neighborhood features; thus, this strategy might ensure optimal measurements of neighborhood safety features.

This study has implications for primary and secondary prevention of adolescent overweight through the development of contextually-relevant interventions and policies. Adolescents in our sample report fear in their neighborhood and high levels of exposure to neighborhood violence, as others [45-49] have shown. This is concerning on its own and also because neighborhood safety may be a factor in adolescent overweight. Our study underscores the importance of policy-level overweight prevention strategies via reducing neighborhood safety concerns. A relevant policy intervention is crime prevention through environmental design [61,62], which would involve changes to the physical environment (such as elimination of hiding spots, landscaping trees and shrubs, and increased surveillance via increased lighting, closed-circuit television/surveillance cameras in public spaces, and/or security guards). Problem-oriented policing, i.e. increased local police attention in "hot spots" or high-crime locations, is another method used to reduce and prevent crime and violence [61,62]. Other potential strategies to reduce neighborhood safety concerns are revitalizing neighborhood watch programs to monitor criminal activity and liaising with police to enhance the protection of places where one can engage in physical activity (e.g. parks and recreation facilities). Furthermore, interventions that offer adolescents' safe havens (such as after-school programs) [63], those that are focused on community development (e.g. ensuring neighborhood resources [such as organizations, services, and employment opportunities]) [64,65], and building collective efficacy among community members [66] could prove beneficial to reduce neighborhood violence. Lastly, it is imperative that behavior change programs (e.g. behavioral weight loss programs) recognize the neighborhood social context of the participants. Physicians, for example, should recommend indoor physical activity for overweight prevention and weight management for adolescents who reside in unsafe neighborhoods.

### Study Limitations

These findings should be interpreted in light of the limitations of our study. First, we relied on cross-sectional data; thus, the study does not inform us about the direction of causation (e.g. whether the exposure preceded the outcome). However, despite the well-known limitations of cross-sectional data, our study hypotheses and directionality have intuitive appeal and were based on conclusions from past research. Additionally, we did not evaluate specific dimensions of neighborhood safety (as previously mentioned) and we did not evaluate objective measures of neighborhood safety (e.g. crime statistics or statistics on the number of sex offenders); we were particularly interested in understanding perceived neighborhood safety rather than the actual occurrence of neighborhood crime or violence. Third, we relied on self-reported height and weight data for BMI, which has the potential for misclassification because of inaccurate reporting. Past research, however, has found that adolescents can provide valid reports of height and weight [67]. Though the gold standard is to collect objectively measured height and weight data, this was not practical nor a central focus of the parent study. Residual confounding might also be a concern, as the survey might have excluded important confounding variables associated with both the independent variable and the dependent variable (e.g. household income, parental education and residential stability might be confounders), but we were unable to account for these variables in the adjusted regression analyses because they were not asked in the BYS data collection instrument. Due to expected high rates of non-response, we did not ask these questions. Finally, this study was conducted in one specific geographically-defined population; thus, these findings might only be generalizable to adolescents in comparable urban locations.

## Conclusion

This study adds to the evidence base that neighborhood safety may be associated with overweight status among urban adolescents in certain racial/ethnic groups. Policies and programs should continue to be implemented to reduce neighborhood safety concerns (such as gang activity and witnessing violence) to prevent adolescent overweight.

## Competing interests

The authors declare that they have no competing interests.

## Authors' contributions

DTD conceived the study, assisted with the statistical analysis, interpreted the results, and drafted the manuscript. RMJ assisted with the study design, performed the statistical analysis, interpreted the results, and assisted with writing the manuscript. BEM and DA coordinated the overall survey implementation and data collection, and critically revised the manuscript for substantial intellectual content. All authors have read and approved the final manuscript.

## Pre-publication history

The pre-publication history for this paper can be accessed here:


